# Prediction of the Leaf Primordia of Potato Tubers Using Sensor Fusion and Wavelength Selection

**DOI:** 10.3390/jimaging5010010

**Published:** 2019-01-09

**Authors:** Ahmed Rady, Daniel Guyer, William Kirk, Irwin R Donis-González

**Affiliations:** 1 Department of Agricultural and Biosystems Engineering, Alexandria University, Alexandria 21545, Egypt; 2 Department of Biosystems and Agricultural Engineering, Michigan State University, East Lansing, MI 48824, USA; 3 Department of Plant, Soil, and Microbial Sciences, Michigan State University, East Lansing, MI 48824, USA; 4 Department of Biological and Agricultural Engineering, University of California, Davis, CA 95616, USA

**Keywords:** potatoes, sprouting, primordial leaf count, hyperspectral imaging, spectroscopy, fusion, wavelength selection, PLSR, interval partial least squares

## Abstract

The sprouting of potato tubers during storage is a significant problem that suppresses obtaining high quality seeds or fried products. In this study, the potential of fusing data obtained from visible (VIS)/near-infrared (NIR) spectroscopic and hyperspectral imaging systems was investigated, to improve the prediction of primordial leaf count as a significant sign for tubers sprouting. Electronic and lab measurements were conducted on whole tubers of Frito Lay 1879 (FL1879) and Russet Norkotah (R.Norkotah) potato cultivars. The interval partial least squares (IPLS) technique was adopted to extract the most effective wavelengths for both systems. Linear regression was utilized using partial least squares regression (PLSR), and the best calibration model was chosen using four-fold cross-validation. Then the prediction models were obtained using separate test data sets. Prediction results were enhanced compared with those obtained from individual systems’ models. The values of the correlation coefficient (the ratio between performance to deviation, or *r*(RPD)) were 0.95(3.01) and 0.9s6(3.55) for FL1879 and R.Norkotah, respectively, which represented a feasible improvement by 6.7%(35.6%) and 24.7%(136.7%) for FL1879 and R.Norkotah, respectively. The proposed study shows the possibility of building a rapid, noninvasive, and accurate system or device that requires minimal or no sample preparation to track the sprouting activity of stored potato tubers.

## 1. Introduction

Recent studies have shown various health-promoting nutritional resources in potato tubers including protein, dietary fibers, minerals, ascorbic acids, anthocyanins, and antioxidants. Moreover, phenolic compounds, contained in the tuber or the peel, are known for their anti-inflammatory and anticarcinogenic effects on human health [1]. Due to the rapid change of lifestyles towards fast food and ready-to-cook meals, the consumption of potatoes in the United States, especially frozen French fries and chips, has shown a significant increase during the last four decades [2]. The U.S. per capita French fry consumption jumped from 12.93 Kg in 1970 to 22.89 Kg in 2017 [2]. Hence, maintaining the appropriate degree of tuber quality during handling and storage operations is a major concern for growers and processors, to preserve a high level of marketability.

Storage significantly affects the chemical composition of tubers and subsequent processed products. Potatoes, as other agricultural commodities, continue to perform several postharvest biological processes, among which respiration represents an important metabolic process that needs to be controlled during storage, to extend the shelf life and reduce the accumulation of sugars [3,4]. Dormancy of potato tubers is the duration after harvest during which tubers will not sprout with the presence of the suitable environmental and biochemical conditions. Dormancy usually lasts from several weeks to months, depending on cultivar and storage conditions [5,6]. Following the dormancy period and with warmer temperatures (10–20 °C), sprouts, i.e., the meristematic regions of the tubers (eyes), begin to grow at a low rate that increases until one sprout dominates others [7]. Sprouting is affected by storage conditions, the cultivar, and the presence of damage. Sprouting has shown a significant impact on the physiological status and age of potatoes during storage [7]. Levels of reducing sugars accumulated during low-temperature storage result in after-frying browning, and excess sucrose content causes improper sweetening flavor of fried products [4]. On the other hand, high levels of reducing sugars and sucrose result in an increase in sprouting [4]. Additionally, unrestrained sprouting results in an increase of respiration rate, which leads into an increase of the sprouting, physiological age, weight loss, and the glycoalkaloid levels that are known to be toxic [8]. Thus, uncontrolled storage sprouting causes a considerable decline in the marketability of raw and subsequent processed potato products.

Various techniques have been used to control or inhibit potato sprouting during storage. Low temperature storage is beneficial for minimizing the sprouting of seed tubers [9]. However, sugar accumulation is an expected consequence of storing potatoes at a low temperature [10]. Chemical inhibitors are commonly applied during storage, including isopropyl N-phenylcarbamate (ICP; propham), isopropyl N-(3chlorophenyl) carbamate (CIPC; chloro-IPC, chloropropham), and maleic hydrazide (MH) [10,11,12]. However, ICP and CIPC cannot be applied on seed potatoes for their irreversible sprouting inhibition [13,14].

Near-infrared (NIR) spectroscopy has been studied for detecting chemical constituents and physical properties of agricultural and food products, in addition to pharmaceutical, textiles, cosmetics, and medicine domains [15]. The utilization of NIR technology in the agricultural domain included the quality evaluation of grains [16,17], fruits, and vegetables [18,19,20]. More specifically, the possibility of using NIR systems on determining several quality attributes of potatoes showed promising results. Such properties include specific gravity [21,22], dry matter [23,24], and sugars [24,25,26]. Rady et al. [25] stated that prediction models of leaf primordia for potato tubers had correlation coefficient (r) values of 0.89 and 0.77 for FL1879 and R.Norkotah, respectively, using a VIS/NIR spectroscopic system in the interactance mode. In the case of the VIS/NIR hyperspectral imaging system, the prediction models yielded *r* values of 0.47 and 0.43 for FL1879 and R.Norkotah, respectively. Jeong et al. [27] investigated the application of VIS/NIR diffuse reflectance spectroscopy (400–2500 nm) for estimating the sprouting capacity of Atlantic and Superior potato cultivars. The authors stated that the sprouting capacity could be evaluated by measuring the weight of sprouts grown under a standard sprouting method. Thus, sprouting capacity was measured based on the weight percentage of sprouts for tubers stored for 30 days in the dark at 20 °C and 90% relative humidity. Results showed a good correlation between lab measurements and predicted sprouting capacity, with *r* values falling between 0.87 and 0.97.

The fusion of data acquired from different electronic sensors has been studied for the potential benefits of improving the prediction models of quality attributes of fruits, vegetables, and food products. The data combined from each individual sensor should, however, provide distinguishing and non-redundant information about the measured property. Consequently, the improvement of prediction and classification models can be feasible. Data fusion can be conducted by either concatenating the features from various sensors, then processing them, or by performing feature selection before combining and processing [28].

The fusion of data obtained by stationary and prototype online hyperspectral imaging systems was conducted by Mendoza et al. [29] to improve the prediction capability of firmness and soluble solid content (SSC) for Golden Delicious (GD), Jonagold (JD), and Red Delicious (RD) apple cultivars. Results showed a significant decrease of the standard error of prediction (SEP) values for firmness by 6.6, 16.1, and 13.7% for GD, JG, and RD, respectively. The values of SEP for SSC decreased for GD, JG, and RD by 11.2, 2.3%, and 3.0, respectively. Mendoza et al. [30] examined the fusion of visible and shortwave NIR spectroscopy (400–1100 nm), spectral scattering obtained from hyperspectral imaging (500–1000 nm), acoustic firmness, and bioyield firmness to assess the firmness and SSC of JG, GD, and RD apple cultivars. In such studies, fused data improved firmness prediction models by reducing SEP values by 14.6, 20.0, and 7.3% for JG, GD, and RD cultivars, respectively. In the case of SSC prediction models, the fusion of spectroscopic and hyperspectral imaging systems showed a decrease in SEP values by as much as 6.0%.

The data fusion approach has also been investigated with other agricultural products. Integrating electronic tongue (e-tongue) and UV-VIS-NIR spectroscopic data has been applied for determining the botanical origin of honey [31]. Ignat et al. [32] studied the fusion of VIS/NIR spectroscopic data with VIS hyperspectral imaging features, relaxation and ultrasonic data, and color measurements for predicting several maturity indices for bell peppers, including dry matter (DM), TSS, osmotic potential (OP), ascorbic acid (AA), total chlorophylls, carotenoids, the coefficient of elasticity for compression (CEc), and the coefficient of elasticity for rapture (CEr). Results illustrated the improvement of the determination coefficient (R^2^) for fused data models. Values of R^2^ increased from 0.93 to 0.95 for DM, 0.93 to 0.96 for TSS, 0.79 to 0.83 for AA, 0.87 to 0.90 for OP, 0.60 to 0.77 for total chlorophylls, 0.92 to 0.96 for carotenoids, 0.55 to 0.63 for CEc, and 0.52 to 0.54 for CEr. Several studies were also conducted to boost the evaluation of various quality attributes for fruits and vegetables using data fusion. Such commodities included bell peppers [33,34], tomatoes [35], apples [36,37,38,39], eggplants [40], peaches [41,42], and oranges [43].

The main objective of this study was to investigate the potential of combining data obtained from hyperspectral imaging and spectroscopic systems for building calibration and prediction models of leaf primordia of potato tubers during storage.

## 2. Materials and Methods

### 2.1. Raw Materials, Sampling, and Measurement of Primordial Leaf Count

Electronic measurements were conducted on Frito Lay 1879 (FL1879) and Russet Norkotah (R.Norkotah) potato cultivars used for chipping and baking, respectively. Samples were obtained from a commercial farm in Southwest Michigan, United States. After discarding defected and deteriorated tubers, samples were cleaned and stored at 7 °C for four weeks for periderm maturation [44]. Sampling was first examined on 20 tubers per cultivar. Tubers were then stored at 7, 10, and 15 °C, and sampled at 20, 80, and 130 days of storage with 60 tubers per cultivar. A total of 200 tubers tested form FL1879 or R.Norkotah were tested. The reason for choosing such storage temperatures was to create a broad distribution of leaf primordia, which increases the reliability of the prediction models. The measurements of primordial leaf count (LC) took place as stated in Rady et al. [45].

### 2.2. Electronic Measurements

Whole tubers were electronically scanned using a VIS/NIR spectroscopic system in the interactance mode and a VIS/NIR hyperspectral imaging in the reflectance mode. To obtain consistent measurements, each tuber was placed such that the light beam struck the middle area of the longitudinal axis. More detailed explanation of the scanning process for either system can be found in Rady et al. [45].

#### 2.2.1. VIS/NIR Interactance System

**Figure 1:**
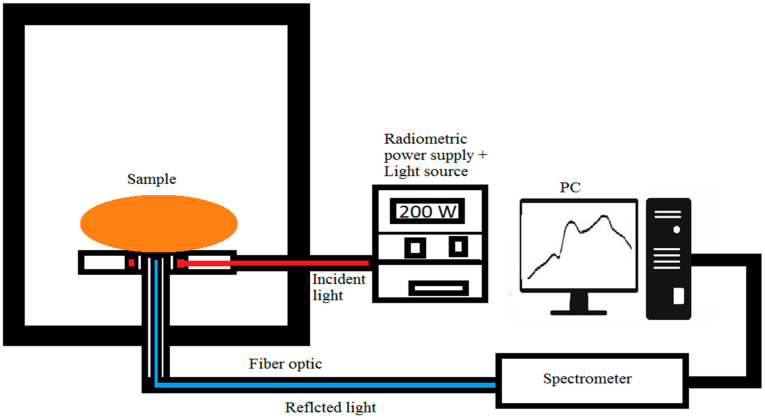
Schematic representation of the visible (VIS)/near-infrared (NIR) interactance testing of Frito Lay 1879 Frito Lay 1879 (FL1879) and Russet Norkotah (R.Burbank) potato cultivars.

The VIS/NIR spectroscopic system in the interactance mode was used to acquire spectral information of the whole tubers. The system, as shown in Figure 1, contained an Ocean optic spectrometer (model No. USB 4000, Ocean Optics, Inc., Dunedin, FL, United States) connected by a 200 μm diameter fiber optic cable, and has a 3648-element linear silicon CCD (charge-coupled device) array with an optical resolution of 0.3 nm (full width half maximum, or FWHM) and a detection range of 200-1100 nm, as well as a radiometric power supply with a maximum power of 250 watts (model No.68931, Oriel Inst., Irvine, CA, United States) and a light source (model No. 66881, Oriel Inst., Irvine, CA, United States) that contained a quartz tungsten halogen lamp and lens transmittance range of 350–2500 nm. In the interactance mode, light photons illuminate the sample through a probe with a concentric outer illumination ring and an inner receptor. A foam-sealing ring was placed between both components for a separation between the light ring and the detector [45]. Thus, only the light passing through the sample was measured. Using such a configuration, the incident light represents a circle with a diameter of 24.7 mm. The interactance spectra for each sample was normalized using a Teflon disc (~25 mm diameter) as a reference material, and the relative interactance was calculated as follows:

$$Relative\;Interactance=\frac{I_{s}-I_{d}}{I_{r}-I_{d}}$$

where *I_s_* is the intensity of the reflected light from the sample, *I_r_* is the intensity of the reflected light from the reference material, and *I_d_* is the intensity of the reflected light from the background.

#### 2.2.2. VIS/NIR Hyperspectral Imaging System 

**Figure 2:**
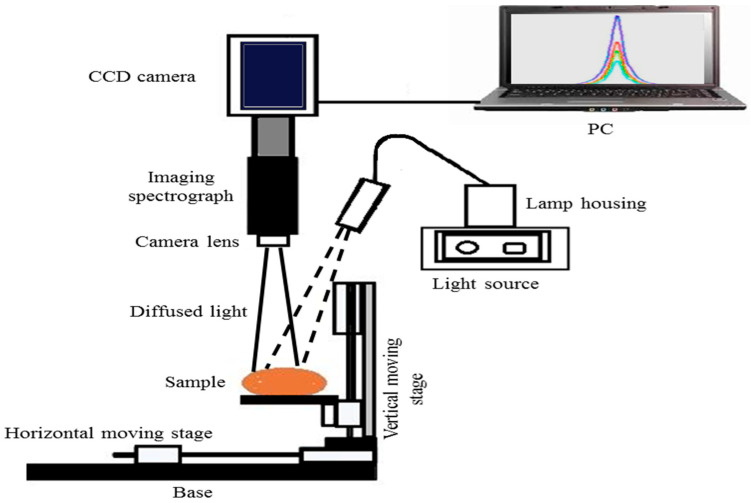
Schematic representation of the VIS/NIR hyperspectral reflectance system used to test whole FL1879 and R.Burbank potato cultivars.

The main target of using a hyperspectral imaging system (HSI) system in this study was to capture the diffuse scattered light in the range of 400–1000 nm under the reflection mode for whole tubers. The system, as shown in Figure 2, contained a Hamamatsu dual mode cooled CCD camera (model No. C4880, Hamamatsu Photonics, Hamamatsu, Japan), an imaging spectrograph directly attached to the CCD camera (ImSpector V10, Spectral Imaging Ltd., Oulu, Finland), a power supply control (model No. 69931, Oriel Instruments Irvine, CA, United States), a digital exposure controller (model No. 68945, Oriel Instruments, Irvine, CA, United States), and a light source (model No. 66881, Oriel Instruments, Irvine, CA, United States) holding a 250 W Quartz Tungsten Halogen lamp and having a lens material transmittance range of 350–2500 nm. A fiber optic cable coupled with a lens focusing assembly was used to deliver a broadband light beam of 1.5 mm diameter, making a 15° angle away from the vertical axis and 1.6 mm apart from the scanning line. The sample holder movement was controlled using a step motor, and each sample was scanned 10 times with a distance of 1 mm between two successive scans, which totally covered the 9 mm longitudinal distance along the sample. The acquisition time was adjusted for each sample at 200 ms, so the total scanning time for each scanning was 2 s. At each scanning line, the spectrograph acquired the spectral information represented by a 256 × 256 pixel image, with spatial and spectral resolutions of 0.2 mm/pixel and 2.35 nm, respectively.

### 2.3. Data Analysis and Fusion

#### 2.3.1. Calculation of the Mean Reflectance Spectra and Wavelength Selection

The average reflectance spectra were calculated for the hyperspectral imaging data, using 256 wavelengths in the range of 400–1000 nm. For each image, the spectra were first averaged over the spatial coordinates. The relative reflectance (RR) spectrum was then calculated as follows:

$$RR=\frac{AS_{s}-AS_{b}}{AS_{r}-AS_{b}}$$

where *AS_s_*, *AS_b_*, and *AS_r_* are the average spectra for the sample, background, and reference (Teflon cube), respectively.

Wavelength selection was conducted to reduce the number of variables involved in multivariate regression, to overcome the possibility of the overfitting problem related to relatively high dimensional data, such as spectroscopic data [46]. Therefore, using wavelength selection techniques improves the robustness of the calibration models and reduces the computational time [47].

Interval partial least squares (IPLS) was adopted as a variable selection technique on the data obtained from spectroscopic and hyperspectral imaging, following the results obtained by Rady and Guyer [48]. The configuration of the applied IPLS included the forward mode, window width (W) of one and two variables, and using 20 latent variables (LV).

#### 2.3.2. Data Fusion

After obtaining the most influencing wavelengths, data from the spectroscopic and hyperspectral imaging systems were normalized at each wavelength (column) by dividing all values at such a wavelength by the maximum value at the same wavelength. For each sample (row), data obtained from both systems was then concatenated to form the fused data matrix.

#### 2.3.3. Partial Least Squares Regression and the Preprocessing of Fused Data 

Partial least squares regression (PLSR) was applied on the fused data to build calibration and prediction models. PLSR is a linear regression technique known for handling high dimensional data and overcoming the colinearity problem associated with such types of data [46].

**Figure 3:**
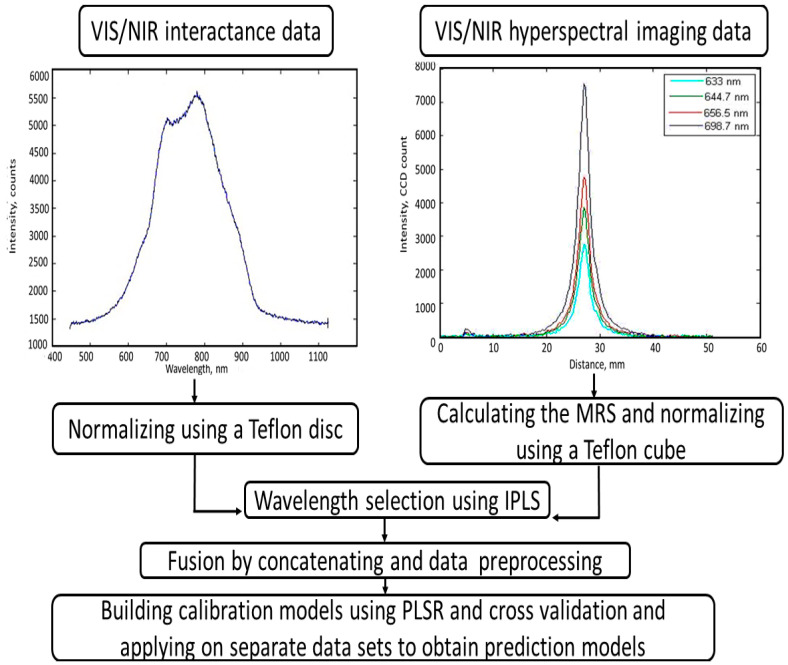
Flow chart of acquiring data from VIS/NIR spectroscopic and VIS/NIR hyperspectral imaging systems, wavelength selection, preprocessing, and building regression models of leaf primordia count for FL1879 and R.Norkotah potato cultivars.

According to Rinnan et al. [49], spectral data contains noisy signals resulting from various electronic sources, and consequently data preprocessing is necessary to reduce such undesirable electronic effects and increase the signal-to-noise ratio. Preprocessing was conducted in two stages. The first stage included, in addition to non-processing, smoothing using a first derivative, smoothing using a second derivative, normalization, a standard normal variate (SNV), multiplicative scattering correction (MSC), and the median center. The second stage included the mean center, multiplicative scattering correction, and orthogonal signal correction. Numerical transformation was also carried out on the reference data (leaf primordia count) to obtain uniform distribution. Logarithmic (base 10) and second degree power transformations were applied, in addition to the non-transformed reference values. The regression analysis was carried out on calibration (80% or 160 tubers) and prediction (20% or 40 tubers) sets of data. To reduce the possibility of overfitting and increase the robustness of calibration models, a four-fold cross-validation technique was implemented on the calibration data set, and the best calibration model was chosen as the one with the minimum root mean square error of calibration for cross validation (RMSEC_cv_). Prediction models were then obtained by applying the optimal calibration models on the separate prediction data sets. A complete layout of the data analysis operations is shown in Figure 3. The best prediction model was chosen based on the values of the correlation coefficient (*r*), the root mean square error of prediction (RMSEP), and the ratio of the standard deviation to the root mean square error of prediction (RPD).

## 3. Results

### 3.1. Constituent Distribution and Wavelength Selection Results 

The minimum, maximum, mean, and standard deviation values of primordial leaf count (LC) were calculated for FL1879 and R.Norkotah cultivars as shown in Table 1. Both cultivars showed close minimum and mean values. Maximum and standard deviation, however, showed higher values in the case of FL1879, which possibly shows more sprouting. The average LC values were 13.47 and 12.96 for FL1879 and R.Norkotah, respectively. Whereas the standard deviation values were 13.62 for FL1879 and 8.61 for R.Norkotah. The high standard deviation values were intentionally conducted using relatively higher storage temperatures to obtain a broad LC range, which helps develop more comprehensive prediction models for LC.

Results of wavelength selection shown in Table 2 indicated that the FL1879 spectral data yielded from the interactance system generally illustrated the highest number of selected wavelengths among all spectral data. In contrast, the number of selected wavelengths obtained from the hyperspectral imaging for R.Norkotah was higher than those obtained from the interactance system, except for W = 2, at which a similar number of wavelengths was selected for both electronic systems. Moreover, the number of selected wavelengths for the hyperspectral imaging was generally higher in the visible spectrum than in the NIR range for both cultivars. In the case of the interactance system, results showed a higher number of selected wavelengths in the NIR range, especially for the R.Norkotah.

### 3.2. Partial Least Squares Regression Results

To make a comparison between the performance of prediction models, based on data obtained from individual or fused sensors, we first illustrate the PLSR results using individual systems data for whole Frito Lay 1879 (FL1879) and Russet Norkotah (R.Norkotah) potato cultivars in Table 3.

**Figure 4:**
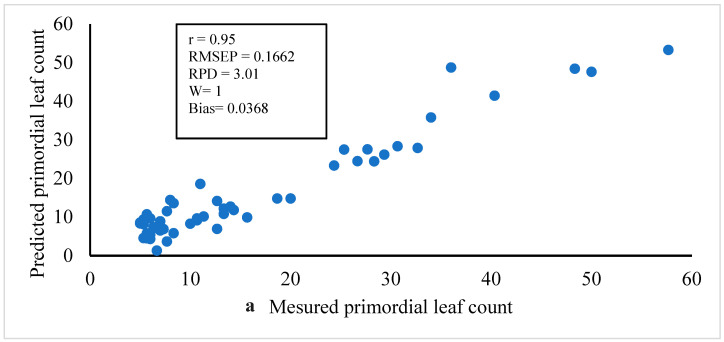

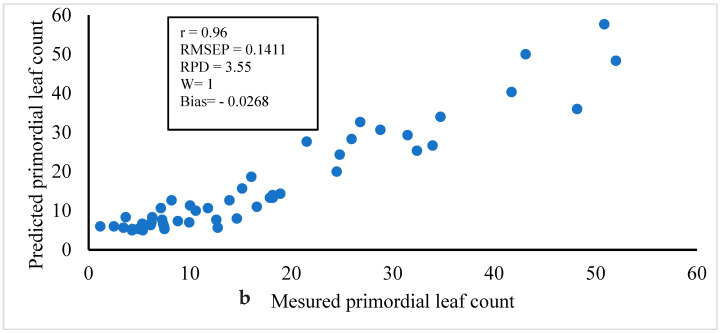
Relationship between measured and predicted primordial leaf count using combined VIS/NIR interactance spectroscopy and VIS/NIR hyperspectral imaging for (**a**) Frito Lay1879 and (**b**) Russet Norkotah.

On the other side, the best PLSR calibration and prediction models of primordial leaf count for FL1879 and R.Norkotah cultivars are shown in Table 4. The optimal models are shown in the shaded cells. In the case of FL1879, the values of *r*(RPD) of prediction models were 0.95(3.01), 0.91(2.27), and 0.91(2.49) for W = 1, 2, and 3, respectively. Whereas, in the case of R.Norkotah, the *r*(RPD) values were 0.96(3.55), 0.95(3.24), and 0.94(2.93), for W = 1, 2, and 3, respectively. The spectral preprocessing methods for the optimal models were first derivative and MSC for FL1879, and second derivative and mean center for R.Burbank. However, the preprocessing of the LC values for the same models was power transformation. The relationship between the measured and predicted LC values for FL1879 and R.Norkotah deduced from the optimal prediction models for W = 1 is shown in Figure 4a,b.

## 4. Discussion

The number of wavelengths selected using the IPLS technique was generally proportional to the window size, especially for R.Norkotah cultivars in the case of data yielded from the two electronic systems; this is expected, as the larger the window size is, the higher the number of selected variables [50]. It was also noted that the interactance data for FL1879 required a higher number of selected wavelengths to explain the variation of LC in comparison to R.Norkotah, except when W = 1 for the hyperspectral imaging. Furthermore, selected wavelengths based on the window width of one variable (W = 1) that were almost the least compared to those obtained using W = 2 or W = 3 yielded the optimal prediction models. Such results illustrate that the small window width could eliminate redundant variables that might be included during the IPLS search algorithm. Zhao et al. [51] developed a modified IPLS method for variable selection, and their study showed a general conclusion that with the low window width, the number of selected variables decreased, and the root mean square error of prediction (RMSEP) improved. Moreover, Deng et al. [52] compared the number of variables selected using different methods, including synergy interval PLS (siPLS), moving window PLS (MWPLS), and genetic algorithm PLS (GA-PLS). Generally, it was obvious that the smaller the window width, the greater the performance of the prediction models.

Using fused data from the two systems, the prediction of LC significantly improved for both cultivars. In a previous study by Rady et al. [25], as shown in Table 1, the optimal prediction models using the VIS/NIR interactance system showed *r*(RPD) values of 0.89(2.22) and 0.77(1.50) for FL1879 and R.Norkotah, respectively. Whereas, the *r*(RPD) values obtained from the VIS/NIR hyperspectral imaging systems were 0.47(1.14) for FL1879 and 0.43(1.10) for R.Norkotah. Additionally, prediction results obtained from the fused data in this study are comparable to the work conducted by Jeong et al. [27] for estimating potato sprouting using NIR diffuse reflectance data. The latter study had *r*(RPD) values of 0.94(2.0) for the calibration models using cross validation. In our study, data fusion led to significant improvement of the prediction performance, which was mainly based on a separate set of data in which the boosted prediction models yielded *r*(RPD) values of 0.95(3.01) and 0.96(3.55) for FL1879 and R.Norkotah, respectively. The fusion of the data, along with wavelength selection, has not been investigated before for the sprouting prediction of potatoes.

The above results indicate that there is a possibility of obtaining a robust prediction of sprouting activity of potato tubers during the storage period, using fused from VIS/NIR spectroscopic and hyperspectral imaging systems. One of the main restrictions of applying hyperspectral imaging systems in on-line sorting and quality inspection processes for food and agricultural products is the relatively long acquisition time. The prediction models obtained in this study, however, were based on selected wavelengths. Thus, decreasing the computation time is accomplished by using fewer wavelengths to build a multispectral imaging system.

## 5. Conclusions

The main objective of this research study was to investigate the potential of utilizing fused data from VIS/NIR spectroscopic and VIS/NIR hyperspectral imaging systems on predicting primordial leaf count of potatoes. Leaf count is an important factor assessing the sprouting capability of tubers; thus, continuous observation of such activity during storage is crucial to maintain the appropriate physiological status of tubers, especially for processing or seeds. Electronic measurements were performed on whole tubers of FL1879 and R.Norkotah potatoes stored at different temperatures, to stimulate the real storage conditions and obtain wide ranges of LC. After obtaining the most influential wavelengths from both electronic systems using IPLS, data from both systems were fused. Results obtained from PLSR indicated a feasible application of the fusion method to considerably improve LC prediction. Compared to the optimal results obtained from individual systems, values of *r*(RPD) have been boosted by 6.7%(35.6%) and 24.7%(136.7%) for FL1879 and R.Norkotah, respectively, which stands as a unique enhancement and application of data fusion for potato sprouting. Results deduced from this study initiate the possibility of developing an electronic system, either portable or stationary, that is composed from multispectral imaging along with an interactance sensors to obtain rapid and accurate prediction of sprouting activity of stored potatoes. However, future steps are still needed to reduce the number of selected wavelengths using different versions of IPLS, such as moving average IPLS, synergy IPLS, backward/forward IPLS, and a genetic algorithm. More cultivars should also be tested, and experiments should be conducted over several growing seasons to improve the robustness and reproducibility of the prediction models.

## Figures and Tables

**Table 1:** Statistical summary of primordial leaf count (LC) measured for Frito Lay 1879 (FL.1879) and Russet Norkotah (R.Norkotah) potato cultivars.

	Minimum	Maximum	Mean	Standard Deviation
FL1879	4.33	57.66	13.47	13.62
R.Norkotah	4.33	45.67	12.96	8.61

**Table 2:** Number of selected wavelengths using the interval partial least squares (IPLS) technique for primordial leaf count, using data obtained from VIS/NIR interactance and hyperspectral imaging systems for Frito Lay 1879 (FL1879) and Russet Norkotah (R.Norkotah) potato cultivars. Shaded cells show optimal models.

		No. of Selected Wavelengths			No. of Wavelengths in the Visible Range			No. of Wavelengths in the NIR Range		
		W = 1	W = 2	W = 3	W = 1	W = 2	W = 3	W = 1	W = 2	W = 3
**VIS/NIR interactance**	**FL1879**	94	106	93	49	40	45	45	66	48
	**R.Norkotah**	26	34	33	1	10	18	25	24	15
**VIS/NIR hyperspectral**	**FL1879**	29	36	63	22	20	39	7	16	24
	**R.Norkotah**	59	34	60	47	21	45	12	13	15

**Table 3:** Partial least squares regression (PLSR) results of the primordial leaf count, using data obtained from either VIS/NIR interactance or hyperspectral imaging systems for Frito Lay 1879 (FL1879) and Russet Norkotah (R.Norkotah) potato cultivars.

Optical System	Cultivar	Calibration *			Prediction **		
		R_cal_	RMSE_CV_	LVs	R_pred_	RMSEP_pred_	RPD_pred_
VIS/NIR interactance system	FL1879	0.99	0.3055	18	0.89	0.3285	2.22
	R.Norkotah	0.91	0.4183	18	0.77	0.3560	1.5
VIS/NIR hyperspectral imaging	FL1879	0.49	13.124	7	0.47	11.7014	1.14
	R.Norkotah	0.78	9.5766	5	0.43	7.8047	1.10

* R_cal_: correlation coefficient for the calibration model; RMSE_cv_: root mean square error of calibration, using cross validation for the calibration model; LVs: number of latent variables. ** R_pred_: correlation coefficient for the prediction model; RMSE_pred_: root mean square error of calibration, using cross validation for the prediction model; RPD_pred_: ratio between standard deviation and the RMSEP_pred_.

**Table 4:** PLSR results for predicting primordial leaf count using data fused from VIS/NIR interactance and VIS/NIR hyperspectral imaging systems for whole tubers for Frito Lay 1879 (FL1879) and Russet Norkotah cultivars. Optimal results are shaded.

Interval Width (W)	Cultivar	Preprocessing Method ^a^	Calibration			Prediction		
			R_cal_	RMSE_CV_	LVs	R_pred_	RMSEP	RPD_val_
W = 1	FL1879	A_5_, B_2_; C_2_	0.99	0.1299	20	0.95	0.1662	3.01
	R.Norkotah	A_6_, B_1_; C_2_	0.98	0.1401	12	0.96	0.1411	3.55
W = 2	FL1879	A_3_, B_1_; C_2_	0.98	0.1815	13	0.91	0.2206	2.27
	R.Norkotah	A_5_, B_2_; C_2_	0.96	0.1775	11	0.95	0.1547	3.24
W = 3	FL1879	A_1_, B_3_; C_2_	0.98	0.1933	20	0.91	0.2012	2.49
	R.Norkotah	A_5_, B_3_; C_2_	0.98	0.1504	17	0.94	0.1709	2.93

^a^ A_x_: First stage spectra preprocessing. A_0_: No preprocessing. A_1_: First derivative. A_2_: Second derivative. A_3_: Normalization. A_4_: Standard normal variate (SNV). A_5_: Multiplicative signal correction (MSC). A_6_: Median center. B_x_: Second stage spectra preprocessing. B_1_: Mean center. B_2_: Multiplicative scattering correction. B_3_: Orthogonal signal correction. C_x_: Reference data preprocessing. C_0_: No reference transformation. C_1_: Log reference transformation. C_2_: Power reference transformation.

## References

[1] Pihlanto A., Mäkinen S., Mattila P., Claudio C. (2012). Potential health-promoting properties of potato-derived proteins, peptides and phenolic compounds. Agriculture Issue and Policies: Production, Consumption and Health Benefits.

[2] United States Department of Agriculture (USDA), Economic Research Services (RRS) (2018). Potatoes: U.S. per capita availability 1970–2017. Vegetables and Pulses Yearbook.

[3] Copp L.J., Blenkinsop R.W., Yada R.Y., Marangoni A.G. (2000). The relationship between respiration and chip color during long-term storage of potato tubers. Am. J. Potato Res..

[4] Stark J.C., Love S.L., Stark J.C., Love S.L. (2003). Tuber Quality, in Potato Production Systems.

[5] Wohleb C.H., Knowles N.R., Pavek M.J., Navarre R., Pavek M. (2014). Plant Growth and Development. The Potato, Botany, Production and Uses.

[6] Cutter E.G. (1992). Structure and development of the potato plant. The Potato Crop The Scientific Basis for Improvement.

[7] Suttle J.C., Vreugdenhil D., Bradshaw J., Gebhardt C., Governs F., Mackerron D.K.L., Taylor M.A., Ross H.A. (2007). Dormancy and sprouting. Potato Biology and Biotechnology Advances and Perspectives.

[8] Pringle B., Bishop C., Clayton R. (2009). Potatoes Postharvest.

[9] Spychalla J.P., Desborough S.L. (1990). Fatty acids, membrane permeability, and sugars of stored potato tubers. Plant Physiol..

[10] Kirk W.W., Davis H.V., Marshall B. (1985). The effect of temperature on the initiation of leaf primordia in developing potato sprouts. J. Exp. Bot..

[11] Afek U., Orenstein J., Nuriel E. (2000). Using HPP (hydrogen peroxide plus) to inhibit potato sprouting during storage. Am. J. Potato Res..

[12] Jedhav S.J., Mazza G., Desai U.T., Salunkhe D.K., Kadam S.S., Jadhav S.J. (1991). Postharvest handling and storage. Potato: Production, Processing, and Products.

[13] Pinhero R.G., Coffin R., Yada Y.R., Singh J., Kaur L. (2009). Post-harvest storage of potatoes. Advances in Potato Chemistry and Technology.

[14] Daniels-Lake B., Olsen N., Delgado H.L., Zink R. (2013). Potato Sprout Control Products to Minimize Sprout Production, NAPPO Science and Technology Documents.

[15] McClure W.F., Ozaki Y., McClure W.F., Christy A.A. (2007). Near-Infrared Spectroscopy in Food Science and Technology.

[16] Fassio A., Fernández E.G., Restaino E.A., La Manna A., Cozzolino D. (2009). Predicting the nutritive value of high moisture grain corn by near infrared reflectance spectroscopy. Comput. Electron. Agric..

[17] Pearson T. (2009). Hardware-based image processing for high-speed inspection of grains. Comput. Electron. Agric..

[18] Suphamitmongkol W., Nie G., Liu R., Kasemsumran S., Shi Y. (2013). An alternative approach for the classification of orange varieties based on near infrared spectroscopy. Comput. Electron. Agric..

[19] Kumar S., McGlone A., Whitworth C., Volz R. (2015). Postharvest performance of apple phenotypes predicted by near-infrared (NIR) spectral analysis. Postharvest Biol. Technol..

[20] Sánchez M., Garrido-Varo A., Pérez-Marín D. (2013). NIRS technology for fast authentication of green asparagus grown under organic and conventional production systems. Postharvest Biol. Technol..

[21] Chen J.Y., Zhang H., Miao Y., Matsunaga R. (2005). NIR measurement of specific gravity of potato. Food Sci. Technol. Res..

[22] Scanlon M.G., Pritchard M.K., Adam L.R. (1999). Quality evaluations of processing potatoes by near infrared reflectance. J. Sci. Food Agric..

[23] Subedi P.P., Walsh K.B. (2009). Assessment of potato dry matter concentration using short-wave near-infrared spectroscopy. Potato Res..

[24] Haase N.U. (2011). Prediction of potato processing quality by near infrared reflectance spectroscopy of ground raw tubers. J. Near Infrared Spectrosc..

[25] Rady A.M., Guyer D.E., Kirk W., Donis-Gonzalez I.R. (2014). The potential use of visible/near infrared spectroscopy and hyperspectral imaging to predict processing-related constituents of potatoes. J. Food Eng..

[26] Yaptenco K.F., Kawakamis S., Takano K. (2000). Nondestructive determination of sugar content in ‘Danshaku’ potato (solanum tuberosum l.) by near infrared spectroscopy. J. Agric. Sci. Tokyo Nogyo Daigaku.

[27] Jeong J.-C., Ok H.-C., Hur O.-S., Kim C.G. (2008). Prediction of sprouting capacity using near-infrared spectroscopy in potato tubers. Am. J. Potato Res..

[28] Manso J.Y. (2008). Sensor Fusion of IR, NIR, and Raman Spectroscopic Data for Polymorph Quantitation of an Agrochemical Compound. Master’s Thesis.

[29] Mendoza F., Lu R., Ariana D., Cen H., Bailey B. (2011). Integrated spectral and image analysis of hyperspectral scattering data for prediction of apple fruit firmness and soluble solids content. Postharvest Biol. Technol..

[30] Mendoza F., Lu R., Cen H. (2012). Comparison and fusion of four nondestructive sensors for predicting apple fruit firmness and soluble solids content. Postharvest Biol. Technol..

[31] Ulloa P.A., Guerra R., Cavaco A.M., Rosa da Costa A.M., Fifueira A.C., Brigas A.F. (2013). Determination of the botanical origin of honey by sensor fusion of impedance e-tongue and optical spectroscopy. Comput. Electron. Agric..

[32] Ignat T., Alchanatis V., Schmilovitch Z. (2014). Maturity prediction of intact bell peppers by sensor fusion. Comput. Electron. Agric..

[33] Mohebbi M., Amiryousefi M.R., Hasanpour N., Ansarifar E. (2011). Employing an intelligence model and sensitivity analysis to investigate some physicochemical properties of coated bell pepper during storage. Int. J. Food Sci. Technol..

[34] Ignat T., Mizrach A., Schmilovitch Z., Fefoldi J., Egozi H., Hoffman A. (2010). Bell pepper maturity determination by ultrasonic technique. Prog. Agric. Eng. Sci..

[35] Baltazar A., Aranda J.I., Gonzalez-Aguilar G. (2008). Bayesian classification of ripening stages of tomato fruit using acoustic impact and colorimeter sensor data. Comput. Electron. Agric..

[36] Li C., Heinemann P., Sherry R. (2007). Neural network and Bayesian network fusion models to fuse electronic nose and surface acoustic wave sensor data for apple defect detection. Sens. Actuators B Chem..

[37] Kavdir I., Guyer D.E. (2003). Apple grading using fuzzy logic. Turk. J. Agric..

[38] Zude M., Herold B., Roger J.M., Bellon-Maurel V., Landahl S. (2006). Nondestructive tests on the prediction of apple fruit flesh firmness and soluble solids content on tree and in shelf life. J. Food Eng..

[39] Steinmetz V., Roger J.M., Molto E., Blasco J. (1999). On-line fusion of colour camera and spectrophotometer for sugar content prediction of apples. J. Agric. Eng. Res..

[40] Saito Y., Hatanaka T., Uosaki K., Shigeto K. Eggplant classification using artificial neural network. Proceedings of the International Joint Conference on Neural Networks.

[41] Natale C.D., Zude-Sasse M., Macagnano A., Paolesse R., Herold B., D’amico A. (2002). Outer product analysis of electronic nose and visible spectra: Application to the measurement of peach fruit characteristics. Anal. Chim. Acta.

[42] Ortiz C., Barreiro P., Correa E., Riquelme F., Ruiz-Altisent M. (2001). Nondestructive identification of woolly peaches using impact response and nearinfrared spectroscopy. J. Agric. Eng. Res..

[43] Steinmetz V., Biavati E., Molto E., Pons R., Fornes I. (1997). Predicting the maturity of oranges with non-destructive sensors. Actae Hortic..

[44] Knowles N.R., Plissey E.S., Johnson D.A. (2007). Maintaing tuber health during harvest, storage, and post-storage handling. Potato Health Management.

[45] Rady A., Guyer D.E., Lu R. (2015). Evaluation of sugar content of potatoes using hyperspectral imaging. J. Food Bioprocess Technol..

[46] Varmuza K., Filmoser P. (2009). Introduction to Multivariate Statistical Analysis in Chemometrics.

[47] Mark H., Burns D.A., Ciurczak E.W. (2001). Data Analysis: Multilinear regression and principal component analysis. Handbook of Near-Infrared Analysis.

[48] Rady A., Guyer D.E. (2015). Utilization of visible/near-infrared spectroscopic and wavelength selection methods in sugar prediction and potatoes classification. J. Food Meas. Charact..

[49] Rinnan Å., Berg F., Engelsen S.B. (2009). Review of the most common pre-processing techniques for near-infrared spectra. Trends Anal. Chem..

[50] Wise B.M., Gallagher N.B., Bro R., Shaver J.M., Windig W., Kock R.S. (2006). PLS_Toolbox 4.0 for Use with Matlab.

[51] Zhao Y., Wang S., Li Z., Pei Z., Cao F. (2016). An improved changeable size moving window partial least square applied for molecular spectroscopy. Chemom. Intell. Lab. Syst..

[52] Deng B.-C., Yun Y.-H., Ma P., Lin C.-C., Ren D.-B., Liang Y.-Z. (2015). A new method for wavelength interval selection that intelligently optimizes the locations, widths and combinations of the intervals. Analyst.

